# Key Genes and Pathways Associated With Inner Ear Malformation in SOX10 ^*p.R109W*^ Mutation Pigs

**DOI:** 10.3389/fnmol.2018.00181

**Published:** 2018-06-05

**Authors:** Qing-Qing Hao, Liang Li, Wei Chen, Qing-Qing Jiang, Fei Ji, Wei Sun, Hong Wei, Wei-Wei Guo, Shi-Ming Yang

**Affiliations:** ^1^Beijing Key Laboratory of Hearing Impairment Prevention and Treatment, Key Laboratory of Hearing Impairment Science, Chinese PLA Medical School, Beijing, China; ^2^Department of Laboratory Animal Science, College of Basic Medical Sciences, Third Military Medical University, Chongqing, China; ^3^Department of Communicative Disorders & Sciences, Center for Hearing and Deafness, State University of New York at Buffalo, Buffalo, NY, United States

**Keywords:** SOX10, miniature pigs, mondini deformity, bioinformatics analysis, ceRNA network

## Abstract

SRY-box 10 (SOX10) mutation may lead to inner ear deformities. However, its molecular mechanisms on inner ear development are not clear. In this work, the inner ear morphology was investigated at different embryonic stages of the SOX10 mutation miniature porcine model with sensorineural hearing loss, and high-throughput RNA-seq and bioinformatics analyses were applied. Our results indicated that the SOX10 mutation in the miniature pigs led to an incomplete partition (IP) of the cochlea, a cystic apex caused by fusion from middle and apical turns, cochlear modiolar defects and a shortened cochlear duct. The model demonstrated 173 differentially expressed genes (DEGs) and 185 differentially expressed long non-coding RNAs (lncRNAs). The down-regulated DEGs most significantly enriched the inflammatory mediator regulation of the TRP channels, arachidonic acid metabolism, and the salivary secretion pathways, while the up-regulated DEGs most significantly enriched the systemic lupus erythematosus and alcoholism pathways. Based on gene cluster analysis, we selected four gene groups: WNT1, KCNQ4, STRC and PAX6.

## Introduction

Approximately 20% of children with congenital deafness have inner ear deformities, among which the Mondini deformity is the most common type of cochlear malformation. Congenital malformations of the inner ear result from interrupted formation of the membranous labyrinth during pregnancy, constituting approximately 20% of congenital sensorineural hearing loss (Jackler et al., 1987; Sennaroglu and Saatci, 2002; Sennaroglu et al., 2006). Based on embryogenesis and radiology, cochlear malformations can be categorized into five groups: complete labyrinthine aplasia (Michel deformity), cochlear aplasia, common cavity, cochlear hypoplasia and incomplete partition (IP; Jackler et al., 1987). IP anomalies of the cochlea can be further divided into two types: IP-I and IP-II (the classic Mondini deformity; Sennaroglu and Saatci, 2002; Sennaroglu et al., 2006). In 2006, a case with a bilateral symmetric IP defect was reported to be similar to cases that were reported as X-linked deafness and was thereby recognized as IP-III (Sennaroglu et al., 2006). The classic Mondini deformity, first reported by Mondini in 1791 and defined as IP-II, is characterized by fusion of the middle and apical cochlear turns, resulting in a cystic apex with a normal basal turn (Chen et al., 2014). The classic Mondini deformity is reported to be the most common type of cochlear anomaly. However, its genetic causes are unknown (Jackler et al., 1987; Aldhafeeri and Alsanosi, 2016).

Hair cells, supporting cells and sensory neurons originate from the otic placode or vesicle. Melanocytes in the stria vascularis and the ganglionar Schwann cells are derived from neural crest cells (NCCs; Magariños et al., 2012; Whitfield, 2015). It was recently suggested that SRY-Box 10 (SOX10) plays an important and independent role in inner ear development. SOX10, an essential transcription factor in neural crest development, belongs to the SOX family of transcription factors that are involved in determining cell fate and developing cell lineage. SOX10 is closely related to SOX8 and SOX9, all of which contain an HMG (high mobility group) DNA binding domain (Southard-Smith et al., 1999; Pingault et al., 2010). SOX10 mutations induce neural crest defects with diverse phenotypes, including several syndromes with sensorineural hearing loss, such as type II Waardenburg syndrome (WS2, depigmentation and deafness, OMIM #611584), type IV Waardenburg syndrome (WS4, depigmentation, deafness and Hirschsprung disease [HD, absence of enteric ganglia in the distal intestine, OMIM #142623], OMIM #613266), PCWH (peripheral demyelinating neuropathy, central dysmyelinating leukodystrophy, WS and HD, OMIM #609136) and Kallmann syndrome (KS, hypogonadism and anosmia, OMIM #308700; Bondurand and Sham, 2013; Pingault et al., 2014). In humans, the SOX10 gene begins expression in the otic vesicle of 4-week-old embryos and continues to present throughout the cochlear duct epithelium of 10-week-old embryos. Its expression is confined to specific cells from the neural crest and supporting cells in the organ of Corti at 20 weeks (Bondurand et al., 1998; Locher et al., 2013). In mice, the SOX10 expression distribution coincides with that of humans, except that SOX10 is widely expressed in the human cochlear duct epithelium, as the neonatal organ of Corti is still maturing (Wakaoka et al., 2013). Statistical analysis has shown that Waardenburg syndrome patients with SOX10 mutations are more likely to have varying degrees of inner ear deformities (Elmaleh-Bergès et al., 2013; Xu et al., 2016). Although accumulating evidence has shown that SOX10 mutations can induce inner ear malformations in humans, the target genes and pathways regulated by SOX10 involved in the inner ear development remain to be fully investigated. Although rodents are the most commonly used animal models, their inner ear morphogenesis is not analogous to that of humans. Miniature pigs are reported to be more similar to humans, in both morphology and development of the inner ear (Guo et al., 2015). A missense mutation (c.325A>T) of SOX10 in the minipig model was previously developed (Zhou et al., 2016). In this study, the miniature pig was used to investigate the molecular mechanisms involved in the inner ear malformations induced by the SOX10 mutation.

## Materials and Methods

### Animals

Generation of the SOX10 missense mutation (c.325A>T) in Chinese Bama-minipigs has been described previously (Zhou et al., 2016). Briefly, this transgenic miniature porcine model has a heterozygous point mutation (c.325A>T) in exon2 of the Sox10 gene that results in a p.Arg109Trp missense mutation of the SOX10 protein. This mutation is orthologous to the SOX10 p.Arg106Trp in humans that was previously reported in WS2 patients (Chaoui et al., 2011). All experimental animals and embryos used in this study were in accordance with approved guidelines of the Institutional Animal Care and Use Committee of the Third Military Medical University (Approval ID: SYXK-PLA-2007036). The experimental protocols were approved by the licensing committee of the Chinese PLA Medical School and the Third Military Medical University.

### Auditory Brainstem Response (ABR) Tests

A total of 30 Chinese Bama miniature pigs (male and female) were used in the auditory brainstem response (ABR) tests, including 15 wild-type (WT) pigs in the control group and 15 SOX10 mutant-type pigs in the experimental group. The ages ranged from 7 days to 30 days postnatal. All minipigs were anesthetized with ketamine (30 mg/kg) and xylazine (0.1 mg/kg). Electrodes were inserted at the vertex and pinna, and a high frequency transducer (Model M014600) was placed approximately 2 cm from the testing ear. ABRs were evoked with clicks and tone bursts (0.5 ms rise/fall time, 30/s repetition rate) at frequencies of 4, 8, 16, 20 and 32 kHz. Intelligent Hearing System (Intelligent Hearing System, Miami, FL, USA) was used to test the pigs’ ABR threshold. The ABR threshold was determined by visual inspection.

### Micro-CT Scan and Three-Dimensional (3D) Reconstruction

To explore the 3D reconstruction of the inner ear, six minipigs were scanned (including 3 WT and 3 mutant-type pigs) and sacrificed with an overdose of urethane (1.5 g/kg). The inner ears were scanned by a micro-CT system (GEHC #RS0800604-0063) and the data were processed by the eXplore Scan Control System. The inner ear structure was reconstructed using Minics 17.0 software.

### Celloidin-Embedded Cochlea Stained With Hematoxylin-Eosin

Embryos at different developmental stages were collected for the morphology analysis of the inner ear, including E23, E26, E29, E38, E45, E56, E63, E73, E84 and E91. The specimens were fixed in 4% formalin for 1 day, decalcified with 10% EDTA, and dehydrated through graded concentrations of alcohol. Next, the tissues were consecutively embedded in 2.5% celloidin for 1 week, 5% for 1 week, and 12% for 2 weeks. The celloidin-embedded specimens were sectioned in the horizontal plane at 5 μm thickness. The sections were stained with hematoxylin and eosin (HE) and examined by standard light microscopy. Details of these processes have been described in our previous publications (Guo et al., 2015).

### RNA Isolation From Otic Tissue

Otic vesicles were obtained from five WT and five mutant embryos at E28. Total RNA was extracted using Trizol reagent (Invitrogen, CA, USA) following the manufacturer’s protocol. The quantity and purity of the collected total RNA were analyzed with a Bioanalyzer 2200 and RNA 6000 Nano LabChip Kit (Agilent, CA, USA). RNA with an RNA Integrity Number (RIN) above 7.0 was used for further study with NGS (next-generation sequencing). Approximately 10 μg of total RNA representing a specific adipose type was used to deplete the ribosomal RNA per the Epicentre Ribo-Zero Gold Kit instructions (Illumina, San Diego, CA, USA).

### Library Construction and Sequencing

The remaining RNA was fragmented into small pieces using divalent cations under an elevated temperature. The NEBNext^®^ Ultra™ Directional RNA Library Prep Kit for Illumina was used for the RNA-seq library preparation. Library sequencing was conducted with an Illumina HiSeq™ 3000 system following the vendor’s recommended protocol (TruSeq^R^RNA Sample Preparation v2 Guide, Illumina Company Ltd., San Diego, CA, USA).

### RNA-Seq Reads Mapping and Transcript Abundance Estimation

All sample reads were aligned to the UCSC^1^ reference genome using the Tophat package, which initially removed a portion of the reads based on quality information from each read and then mapped the reads to the reference genome. Tophat allowed multiple alignments of the PE reads (up to 20 by default) and a maximum of two mismatches when mapping the reads to the reference. A database of potential splice junctions was built and confirmed by comparing the previously unmapped reads against the database of putative junctions. The aligned read files were processed by Cufflinks, which used the normalized RNA-seq fragment counts to measure the relative abundances of the transcriptome. The unit of measurement was fragments per kilobase of exons per million fragments mapped (FPKM). To compare gene expression between two samples, we converted the FPKM to transcripts per million (TPM) using the following formula: TPM = FPKM × 10,00,000/(sum of FPKM). The reference GTF annotation file used in Cufflinks was downloaded from the UCSC database. The raw data was deposited to GEO database (the accession number is GSE110103).

### Differentially Expressed Genes and lncRNAs

Gfold v1.1.2 was used to count the number of reads mapped to each gene. Differential expression was assessed by DESeq using FPKM as input. Differentially expressed genes (DEGs) and lncRNAs (DE lncRNAs) were chosen by the criteria of at least a 2-fold change and adjusted *P*-value < 0.01.

### Gene Ontology (GO) and Pathway Enrichment Analysis of DEGs

To analyze high-throughput genome and transcriptome data, Gene Ontology (GO; Ashburner et al., 2000) and KEGG (Kanehisa et al., 2017) analyses were used. These are common methods of annotating gene products, identifying characteristic biological attributes and linking pathway information. The DAVID database^2^ is an essential online tool for these analyses (Dennis et al., 2003). We uploaded the DEGs list to the DAVID online tool and *P* < 0.05 was considered statistically significant.

### Protein-Protein Interaction (PPI) Network and Module Analysis

To evaluate the interactive relationships among the DEGs, the DEGs were mapped to the Search Tool for the Retrieval of Interacting Genes (STRING) database, and a minimum combined score >0.4 was selected as a significant interaction to be validated experimentally. Protein-protein interaction (PPI) networks were constructed by the Cytoscape software using the molecular complex detection (MCODE) plug-in to screen the modules in the PPI network. The criteria were set as follows: MCODE scores >3, number of nodes >4, and *P* > 0.05 were considered significant.

After the target genes were selected, the transcription binding sites were predicted using the online JASPAR datasets, with the relative profile score threshold at >80%^3^.

### Construction of the ceRNA Network

The porcine miRNA symbols and sequences were obtained from mirBase^4^. The putative lncRNA-miRNA-target gene interactions were evaluated using the Targetscan version 6.0 algorithms^5^. High-confidence miRNA–lncRNA pairs had a Targetscan context score >95 and a miRNA–target gene-pair score >82. These results were imported into Cytoscape software version 3.4.0 to construct a regulatory network.

## Results

### Depigmentation and Profound Sensorineural Hearing Loss in the SOX10 Mutation Miniature Porcine Model

**Figure 1:**
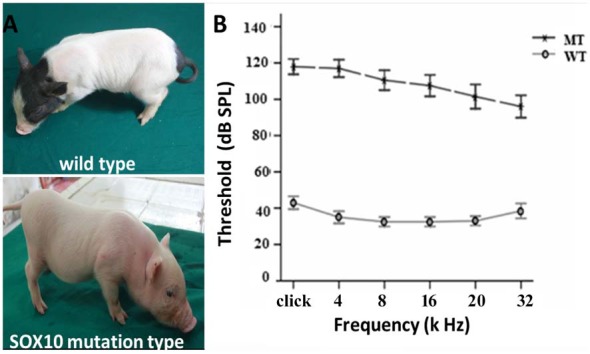
Depigmentation and profound sensorineural hearing loss in the SRY-box 10 (SOX10) mutation miniature porcine model. **(A)** Gross image of a WT bama miniature pig and a mutation-type albino pig. **(B)** The results of the auditory brainstem response (ABR) tests showed profound hearing loss in the SOX10 mutation pigs. The ABR tests showed an absence of recognizable ABR waveforms up to a 110 dB sound pressure level (SPL) with a stimulus range from 4 kHz to 32 kHz in the SOX10 mutation minipigs. The WT minipigs had ABR thresholds at 30–40 dB SPL (WT, wild-type; MT, mutation type; SOX10 mutation-type).

The depigmentation phenotype of the transgenic SOX10 mutation miniature porcine model has been studied for its albinism (Figure 1A) and its similarity to the WS2 phenotype (Zhou et al., 2016). ABR test results showed an absence of recognizable ABR waveforms up to a 110 dB sound pressure level (SPL) with a stimulus range of 4–32 kHz in the SOX10 mutation minipigs, whereas the WT minipigs had ABR thresholds of 30–40 dB SPL (Figure 1B). The ABR tests confirmed that the minipigs with SOX10 mutations developed profound sensorineural hearing loss.

### Inner Ear Phenotype of the SOX10 Missense Mutation Minipigs

**Figure 2:**
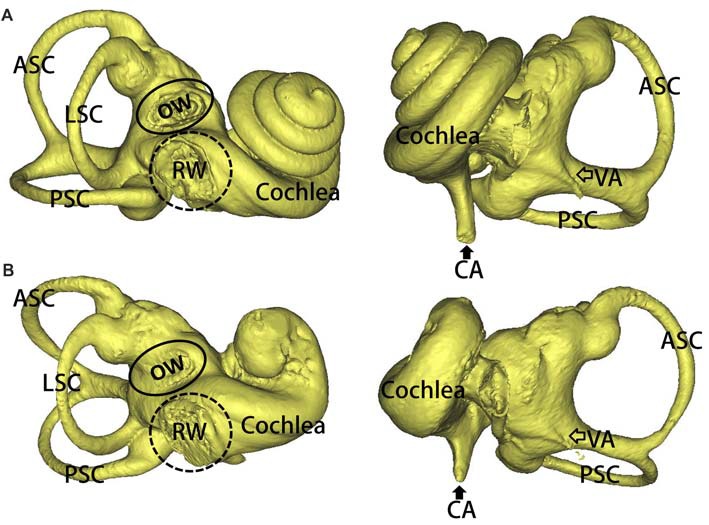
3D reconstruction of the inner ears in the mutation minipigs. **(A)** 3D reconstruction of the inner ear (right) in a WT minipig, anterior and posterior views, respectively. **(B)** 3D reconstruction of the inner ear (right) in a SOX10 mutation minipig, anterior and posterior views, respectively (ASC, anterior semicircular canal; PSC, posterior semicircular canal; LSC, lateral semicircular canal; OW, oval window; RW, round window; VA, vestibular aqueduct; CA, cochlear aqueduct).

Three-dimensional reconstructions of the inner ear, including a WT and a SOX10 MT, are shown in Figure 2. Three semicircular canals in the miniature pigs are perpendicular to each other. The coiling of the cochlea capsule in the WT pigs reached three and one-half turns, while the MT only reached one and a half turns. No apparent enlarged vestibular or cochlear aqueducts (CAs) were detected in the MT compared with the WT.

**Figure 3:**
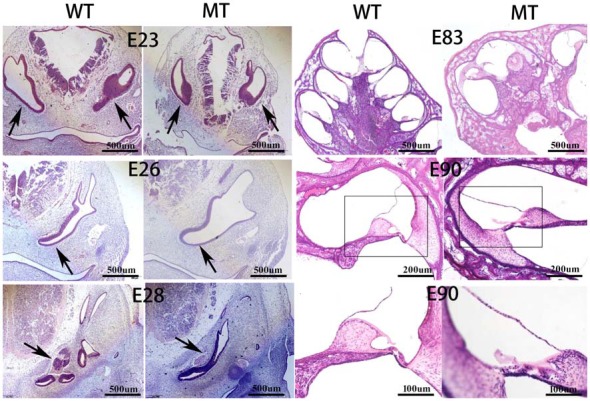
Celloidin sections of the embryonic cochlea stained with hematoxylin and eosin (H & E; MT, mutation type; WT, wild type). The figure illustrates the inner ear morphogenesis of the minipigs at different embryonic stages. The embryonic cochlear celloidin sections were stained with H & E. The otic vesicles (black arrows) were clearly formed at E23 and the cochlear anlagen were observed at E26 in both the WT and SOX10 MT pigs. The E28 cochlear duct (black arrows) lengthened its base by approximately two turns in the WT pig, while it was only one turn in the MT pig. At E83, the WT cochlea were elongated by 3.5 turns, while the MT only had 1.5 turns. At E90, the cochlear development in the WT was almost complete with the mature organ of Corti. In the MT, the cochlear duct remained shorter than the WT, by approximately 1.5 turns with an incomplete partition (IP) leading to the middle and apical turns fusing into a cystic apex. The basal turn was likely to be normal with spiral ganglion cells in the basal part of the modiolus.

Figure 3 illustrates the inner ear morphogenesis of the minipigs at different embryonic stages, including E23, E26, E28, E83 and E90. Celloidin sections of embryonic cochlea were stained with HE. The otic vesicles were clearly formed at E23 and the cochlear anlagen were observed at E26 in both the WT and SOX10 MT pigs. The E28 cochlear duct had a lengthened base of approximately two turns in the WT pigs, while it only had one turn in the MT pigs. At E83, the WT cochlea were elongated to 3.5 turns, while the mutation-type only had 1.5 turns. At E90, cochlear development in the WT pigs was almost complete, with maturing organs of Corti. In the MT, the cochlear duct remained shortened compared to the WT for approximately 1.5 turns and was accompanied by an IP leading to the middle and apical turns, fusing into a cystic apex. However, the basal turn was likely to be normal with spiral ganglion cells in the basal part of the modiolus.

### Identification of Differentially Expressed Genes and lncRNAs

**Figure 4:**
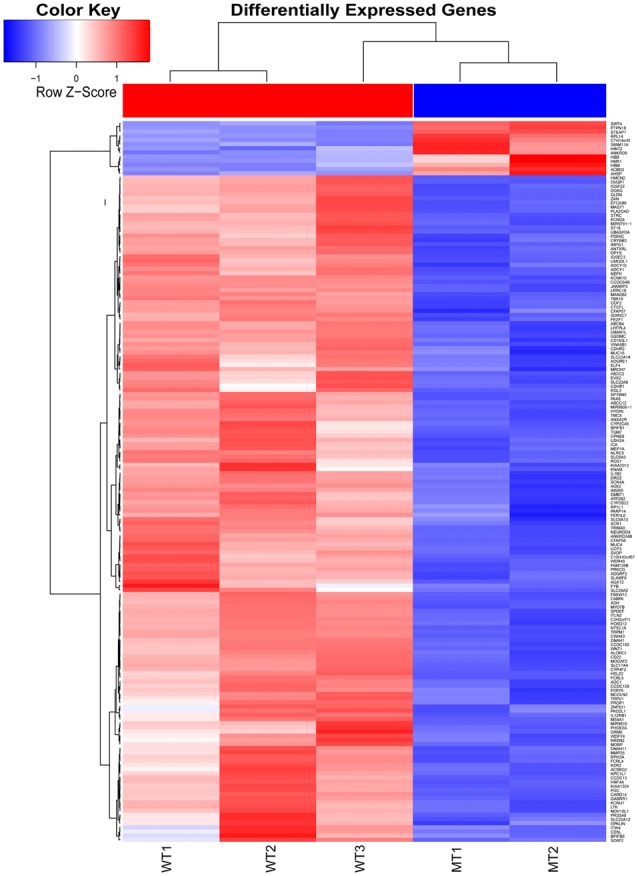
Heat map of differentially expressed genes in the otic vesicles between the two groups at E28. Inner ear samples from five WT and five SOX10 mutation-type pigs at E28 were analyzed. A total of 173 differentially expressed genes (DEGs) were identified. Thirteen DEGs were up-regulated and 160 DEGs were down-regulated in the mutation group (MT, mutation type; WT, wild type; Red, up-regulated; Blue, down-regulated).

**Figure 5:**
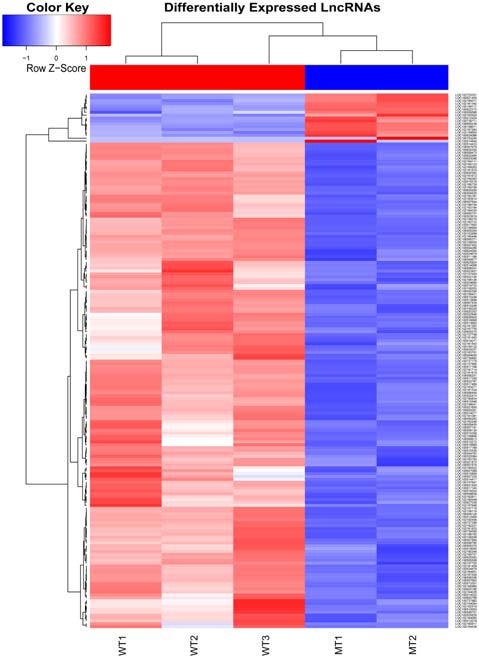
Heat map of differentially expressed lncRNAs in the otic vesicles between the two groups at E28. Inner ear samples from five WT and five SOX10 MT pigs at E28 were analyzed. A total of 185 differentially expressed long non-coding RNAs (DE lncRNAs) were identified. Seventeen DE lncRNAs were up-regulated and 168 DE lncRNAs were down-regulated in the mutation group (MT, mutation type; WT, wild type; Red, up-regulated; Blue, down-regulated).

The inner ear samples of five WT and five SOX10 MT pigs at E28 were analyzed. From the high-throughput RNA-seq (RNA sequencing) data, two samples from the WT group and three samples from the mutation group were excluded due to large intraclass disparities. The criteria used were *P* < 0.05 with a 2-fold change. A total of 173 differentially expressed genes (DEGs) and 185 differentially expressed long non-coding RNAs (DE lncRNAs) were identified. Thirteen DEGs and 17 DE lncRNAs were up-regulated, while 160 DEGs and 168 DE lncRNAs were down-regulated in the mutation group. The hierarchical clustering analyses (heatmaps) of the DEGs and DE lncRNAs (top 100 differentially expressed genes and lncRNAs) are shown in Figures 4, 5.

### Gene Ontology (GO) and Pathway Enrichment Analyses of DEGs

The list of DEGs was uploaded to the online software, DAVID, to analyze the enriched GO categories and KEGG pathways. Based on GO biological processes (BP), the down-regulated DEGs significantly enriched the xanthine catabolic process, forebrain anterior/posterior pattern specification, and retinal development in the camera-type eye, while the up-regulated DEGs enriched ion transport and iron ion homeostasis. Per the cellular components (CC) analysis, the down-regulated DEGs significantly enriched the integral membrane component, the integral plasma membrane component and the newly growing cell tip, while the up-regulated DEGs enriched the hemoglobin complex and the endosome membrane. Based on molecular function (MF), the down-regulated DEGs significantly enriched iron ion binding, oxidoreductase activity (acting on paired donors), and ATPase activity (coupled to transmembrane movement), while the up-regulated DEGs enriched oxygen transporter activity and oxygen binding.

**Figure 6:**
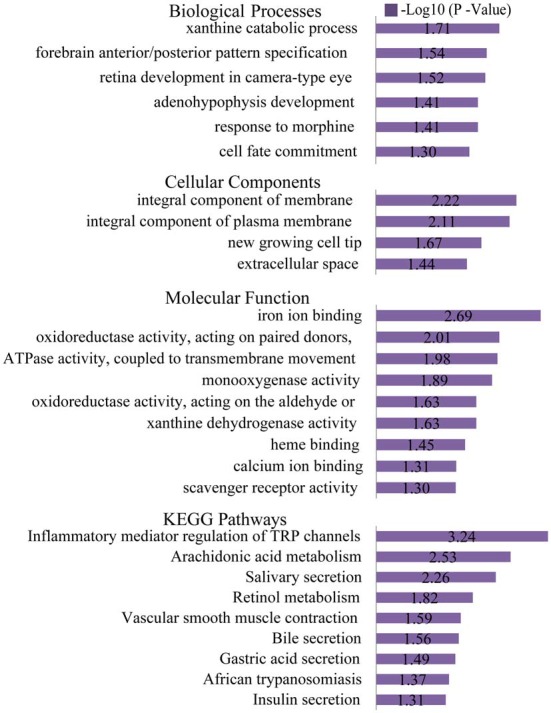
Gene ontology (GO) and KEGG pathway analyses of the down-regulated genes. Based on GO biological processes (BP), the down-regulated DEGs significantly enriched the xanthine catabolic process, forebrain anterior/posterior pattern specification, and retinal development in the camera-type eye. Per the cellular components (CC) analysis, the down-regulated DEGs significantly enriched the integral membrane component, integral plasma membrane component and the newly growing cell tip. Based on the molecular function (MF), the down-regulated DEGs significantly enriched iron ion binding, oxidoreductase activity (acting on paired donors), and ATPase activity (coupled to transmembrane movement). The most significantly enriched pathways associated with the down-regulated DEGs were the inflammatory mediator regulation of TRP channels, arachidonic acid metabolism and salivary secretion.

**Figure 7:**
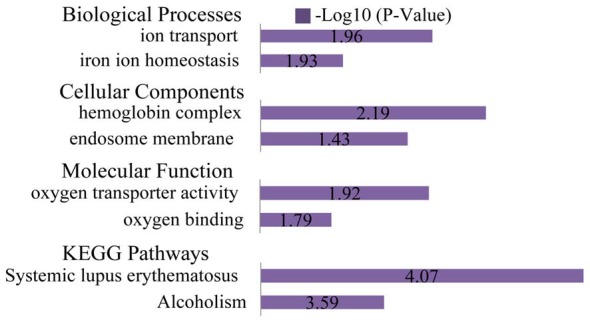
GO and KEGG pathway analyses of the up-regulated genes. Based on GO BP, the up-regulated DEGs enriched ion transport and iron ion homeostasis. Per the CC analysis, the up-regulated DEGs enriched the hemoglobin complex and endosome membrane. Based on the MF, the up-regulated DEGs enriched oxygen transporter activity and oxygen binding. The most significantly enriched pathways associated with the up-regulated DEGs were systemic lupus erythematosus and alcoholism.

The most significantly enriched pathways associated with the down-regulated DEGs were the inflammatory mediator regulation of the TRP channels, arachidonic acid metabolism, and salivary secretion, while those of the up-regulated DEGs were the systemic lupus erythematosus and alcoholism pathways (Figures 6, 7).

### Cluster Analysis to Screen PPI Networks Related to SOX10

**Figure 8:**
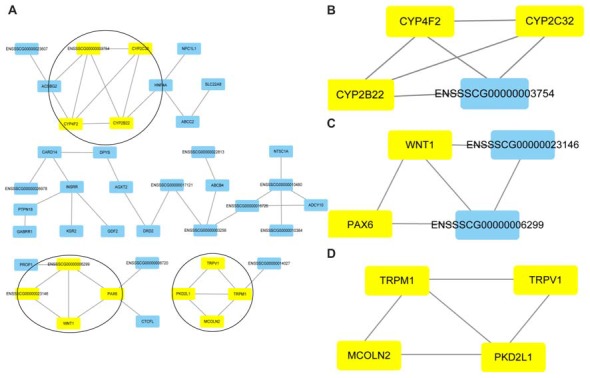
Protein-protein interaction (PPI) networks and the top three clusters of differentially expressed genes. Cluster analyses were performed to screen the PPI networks using the Cytoscape molecular complex detection (MCODE) plug-in based on the STRING database. The figure illustrates the PPI networks of the DEGs based on the STRING database **(A)**. The top three clusters identified by MCODE in the PPI network are shown, respectively **(B–D)**.

Cluster analyses were performed to screen the PPI networks using the Cytoscape MCODE (molecular complex detection) plug-in based on the STRING database. Figure 8 illustrates the DEG’s PPI networks based on the STRING database. Table 1 shows the top three significant clusters that were selected.

**Table 1:** The selected top three significant clusters.

Cluster	Score	Nodes	Edges	Gene IDs
1	4	4	6	CYP2B22, CYP2C32, CYP4F2, ENSSSCG00000003754
2	3.33	4	5	WNT1, PAX6, ENSSSCG00000006299, ENSSSCG00000023146,
3	3.33	4	5	PKD2L1, TRPV1, MCOLN2, TRPM1

**Figure 9:**
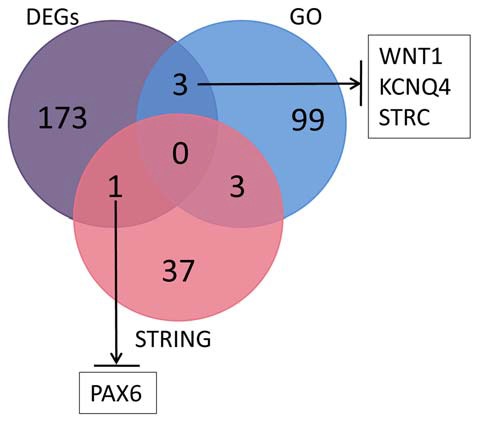
Venn diagram of the intersection among DEGs, GO and the STRING database. We searched the genes related to inner ear development in the GO database and the proteins that interact with SOX10 in the STRING database. We selected four candidate genes: WNT1, KCNQ4, STRC and PAX6. The figure shows the Venn diagram of this intersection (GO, inner ear development related genes in GO; STRING, proteins interacting with SOX10 in STRING; black arrows, down-regulated target genes).

We searched the genes related to inner ear development in the GO database (GO term: inner ear development) and proteins that interact with SOX10 in the STRING database. The gene lists were intersected with the DEGs to obtain the target genes regulated by SOX10 that were associated with inner ear development. We reduced the list of candidate genes to four: WNT1, KCNQ4, STRC and PAX6. Figure 9 shows the Venn diagram of this intersection.

We then predicted the SOX10 binding sites in the candidate genes through the online JASPAR database. Binding sites were found for all candidate genes except for KCNQ4. Table 2 provides the predicted binding site details.

**Table 2:** The SRY-box 10 (SOX10) binding sites predicted in the candidate genes.

Gene	Score	Relative score	Start	End	Strand	Predicted binding site sequence
WNT1	8.63	98.74%	3	8	+	cattgt
STRC	6.35	88.64%	44	49	+	tattgt
STRC	6.81	90.69%	69	74	+	cagtgt
PAX6	4.67	81.17%	88	93	+	ctttgc

### lncRNA–miRNA–Target Genes for ceRNA Network Construction

**Figure 10:**
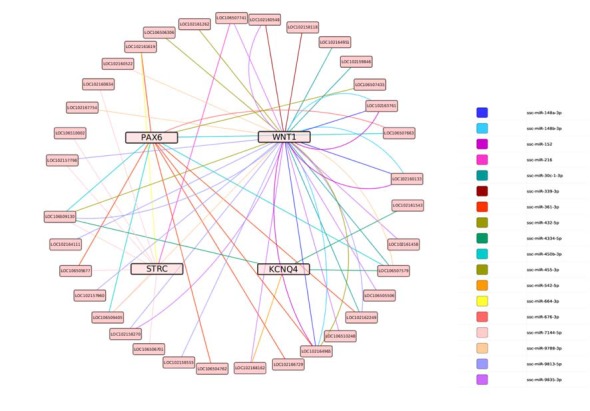
The predicted ceRNA network for the target genes. Lines represent shared miRNAs; pink nodes represent target genes; light pink nodes represent lncRNAs.

Based on this data, the predicted ceRNA network (lncRNA-miRNA-target genes) was constructed using Targetscan 6.0. The regulation network showed that nine miRNAs share 37 lncRNAs with WNT1, two miRNAs share four lncRNAs with KCNQ4, three miRNAs share nine lncRNAs with STRC, and four miRNAs share 132 lncRNAs with PAX6 (as shown in Figure 10).

## Discussion

We studied the phenotypes of previously described miniature pig models with a SOX10 missense mutation. This animal model was shown to be ideal for studying the phenotypes of albinism, profound sensorineural hearing loss and Waardenburg syndrome. SOX10 may be responsible for human Mondini dysplasia, and this strain represents the first inherited animal model for this disease (Hai et al., 2017).

To explore the role of SOX10 on inner ear development, we studied the inner ear morphology using this model. Miniature pigs with the SOX10 mutation provided a good model of the classic Mondini deformity in humans, which is characterized by an IP of the cochlea, a cystic apex caused by fusion from middle and apical turns, cochlear modiolar defects and a shortened cochlear duct (Sennaroglu, 2010). A complete agenesis of the labyrinth in WS patients was previously thought to be a secondary process called cochleosaccular degeneration due to the absence of cranial NCCs, which induced endolymphatic collapse and a secondary defect of the organ of Corti (Elmaleh-Bergès et al., 2013; Locher et al., 2015). Histopathological study of the different embryonic stages in the miniature porcine model demonstrated that the inner ear malformation in WS patients that resulted from the SOX10 mutation was a primary aplasia. This is consistent with the hypothesis that the SOX10 mutation directly induces inner ear malformation rather than being the result of an NCC defect, and the NCCs migrated and differentiated into melanocytes in the intermediate cell layer of the stria vascularis.

To investigate the molecular mechanism of SOX10 on inner ear development, we performed a high-throughput RNA-seq assay on inner ear tissue from the E28 porcine model with the Mondini deformity, as E28 was the onset of inner ear malformation based on the celloidin sections. Compared with the WT pigs at the same embryonic stage, a total of 173 differentially expressed genes and 185 lncRNAs were identified. Among these, 160 DEGs (92.5%) and 168 lncRNAs (90.8%) were down-regulated. Transcription factor SOX10 contained a highly conserved HMG domain that could bind to the target gene to regulate its transcription (Southard-Smith et al., 1999; Pingault et al., 2010). Hypothetically, mutations in SOX10 located upstream of the HMG domain may result in haploinsufficiency (Bondurand et al., 2007; Ruiz-Maldonado, 2007; Zhang et al., 2012). In this study, most of the down-regulated genes and lncRNAs appeared to be a result of the mutation site located at the beginning of HMG domain.

Per the enrichment analyses of the GO and KEGG pathways for the DEGs, we found that the biological process focused on differentiating the anterior-posterior axis involving WNT1, PAX6 and FEZF1, and the cell components enriched the cell membrane, primarily in the ion channel. This suggests that defective cell differentiation functions along the anterior-posterior axis might cause cochlear malformation, and ion transport in the defective cochlear cells may be substantially altered from normal cells. This is inconsistent with previous reports on congenital malformations, which state that inflammation mediated pathways are the main reason for acquired hearing loss caused by infection (Smith et al., 2005).

We also conducted a cluster analysis to screen the PPI network related to SOX10, and ascertained four candidate genes that may be regulated by SOX10 in inner ear development: WNT1, KCNQ4, STRC and PAX6. Furthermore, the SOX10 binding sites were predicted in WNT1, STRC and PAX6. In the last decade, cytoplasmic lncRNAs were found to be responsible for post-transcriptional regulation through their ability to compete for miRNA binding sites (called ceRNAs), which can sequester miRNAs and subsequently protect their target mRNAs from repression (Chen, 2016; Rashid et al., 2016). Therefore, we predicted and constructed the ceRNA network for these four target genes; however, the regulatory mechanism of these ceRNAs requires further study.

**Figure 11:**
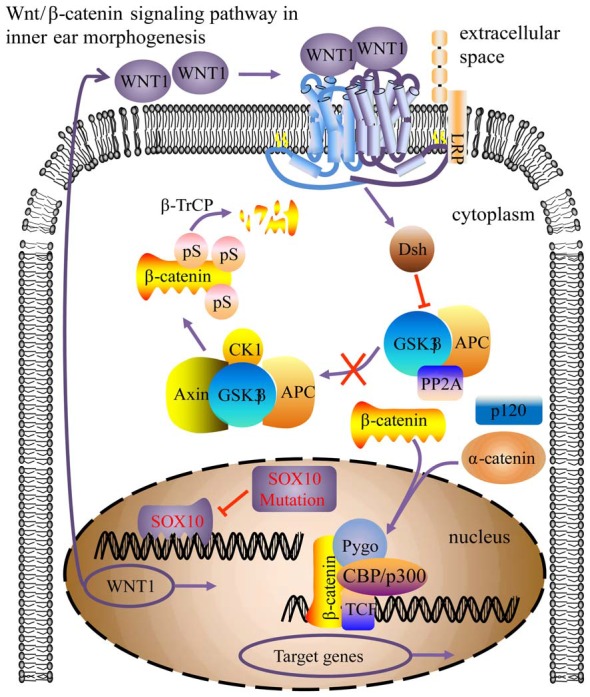
Model for the SOX10-regulated Wnt/β-catenin pathway in inner ear morphogenesis. The SOX10 mutation modified its protein conformation, inducing down-regulation of WNT1 (CBP/p300, Pygo, TCF activator proteins; Dsh, repressor protein; arrow indicates pathway activation; blunt arrow indicates pathway repression).

WNT1 is part of the Wnt/β-catenin signaling pathway, which is involved in inner ear development processes, including otic placode induction, vestibular and cochlear morphogenesis, and prosensory progenitor specification (Riccomagno et al., 2005; Jacques et al., 2012; Forristall et al., 2014; Brown et al., 2015; Chen, 2016). Impaired cochlear development in Wnt1^−/−^ embryos results from defective Wnt signaling activity at the origination stage of the cochlea (Smith et al., 2005; Brown et al., 2015). This study found that WNT1 may be a crucial target gene for SOX10 during inner ear development, particularly during morphogenesis. Due to the HMG domain mutation inducing conformational change, the transcription factor SOX10 failed to bind with the WNT1 gene and consequently down-regulated WNT1 expression. Consequently, the Wnt/β-catenin signaling pathway activity was impaired, leading to compromised cochlear morphogenesis (Figure 11). In addition, mutations in KCNQ4 (Gao et al., 2013; Wang et al., 2014) and STRC (Francey et al., 2012) can induce sensorineural deafness, but this has not been found with PAX6.

In conclusion, high-throughput transcriptome sequencing and bioinformatic analysis of the inner ear malformation in the SOX10^p.R109W^ mutation miniature porcine model provides potential targets for investigating the molecular mechanisms of SOX10 on inner ear development. However, further research is required to confirm the roles of the identified target genes and their pathways in inner ear malformation.

## Author Contributions

S-MY, W-WG and HW conceived and designed the study. Q-QH and LL performed the experiments. Q-QH, LL, WC and Q-QJ wrote the article. WC, Q-QJ, FJ and WS reviewed and edited the manuscript. All authors read and approved the manuscript.

## Conflict of Interest Statement

The authors declare that the research was conducted in the absence of any commercial or financial relationships that could be construed as a potential conflict of interest.

## Footnotes

^1^http://genome.ucsc.edu/

^2^https://david.ncifcrf.gov/

^3^http://jaspar.genereg.net/cgi-bin/jaspar_db.pl

^4^http://mirtarbase.mbc.nctu.edu.tw/

^5^http://www.targetscan.org/
